# Chemical properties of the coffee grounds and poultry eggshells mixture in terms of soil improver

**DOI:** 10.1038/s41598-022-06569-x

**Published:** 2022-02-16

**Authors:** Barbara Tombarkiewicz, Jacek Antonkiewicz, Marcin W. Lis, Krzysztof Pawlak, Magdalena Trela, Robert Witkowicz, Olga Gorczyca

**Affiliations:** 1 Department of Zoology and Animal Welfare, University of Agriculture in Cracow, Cracow, Poland; 2 Department of Agricultural and Environmental Chemistry, University of Agriculture in Cracow, Cracow, Poland; 3 E.G.G. Ltd. L.P., Moniuszki 15, 42-672 Wieszowa, Poland; 4 Institute of Plant Production, University of Agriculture in Cracow, Cracow, Poland

**Keywords:** Biogeochemistry, Environmental sciences

## Abstract

Spent coffee grounds (SCG) as well as chicken (CES) or duck eggshells (DES) left over from the artificial hatching technology are proposed as potential soil improver and/or organic-mineral fertiliser components. Therefore, it seems interesting and necessary to evaluate the chemical composition of these wastes and their mixtures in terms of their possible use for that purpose. The study was conducted under the incubation experiment conditions using a mixture of SCG and eggshells (10:1 ratio). Macronutrients, i.e. C, N, S, were determined by the catalytic combustion method, while P, K, Mg, Ca, Na by atomic spectrometry. It was found that SCG were rich in C, N, P, and K, while eggshells in Ca, Mg, Na, and S. However, CES compared to DES were richer in deacidifying components (i.e. Ca, Mg, K). At the same time, the content of macronutrients in eggshells decreased gradually along with the embryo development. For this reason, the mixture of SCG and shells of unembryonated chicken eggs (CES I) had the best chemical and usable proprieties. To conclude, the chemical properties of the mixtures of spent coffee grounds and eggshells indicate their possible application in soil bioengineering.

## Introduction

In the era of civilisation, economic and technological development, more and more mineral and organic waste is generated, which should be environmentally managed as part of the circular economy^1,2^. Spent coffee grounds are an example of organic waste for environmental management. This waste should be a good alternative for organic-mineral fertilisers or soil conditioners, allowing to improve physicochemical properties of soil and maintain its fertility. This is because of the substantial content of organic matter and valuable nutrients^3^. Moreover, spent coffee grounds are especially rich in sugars and protein, and are a source of organic carbon and nitrogen^4,5^. They also contain bioactive compounds such as caffeine that can affect microorganisms, thus diminishing the soil’s capacity to release nitrogen^4^.

Chicken and duck eggshells are another waste with different physicochemical properties. Macroscopically, chicken eggshell consists of three main structures: egg white (58–63%), egg yolk (27.5–31%) and shell (9.5–11%)^6^. At the molecular level, the main constituents include water (75%), proteins (12%), lipids (12%), and minerals^6,7^. Chicken and duck eggshells were also identified to have amino acids, which can be a source of N for plants, after applying this waste to soil^8^. Egg shell consists mainly of minerals (primarily calcium, but also boron, chromium, copper, iron, iodine, manganese, sulphur, selenium, silicon, and zinc) occurring in inorganic compounds^9^. Bird eggshells are also a rich source of phosphorus in the form of calcium phosphates that are used for the production of mineral fertilisers^10^, also in implantation materials^8^. Due to their considerable nutrient richness, chicken and duck eggshells can be an alternative fertilisation material for plants, provided they are properly treated and refined^11,12^).

The aim of this study was to determine the chemical composition of spent coffee grounds and chicken and duck eggshells and their mixtures in terms of assessing their possible use for the production of organic-mineral fertilisers or soil improver. Mixing these two types of waste, which differ in their physical and chemical properties, into a homogeneous product, can be an alternative for organic-mineral fertilisers intended for crops with high nutritional requirements. The new alternative deacidifying agent, apart from carbon and calcium, will contain organo-mineral bonds, particularly dedicated to soils with low carbon content. The supply of carbon to soil microorganisms and the provision of valuable macronutrients to plants will be an advantage of the new fertiliser product.

## Materials and methods

The study on determining and assessing the content of nutrients in mixtures of spent coffee grounds with chicken and duck eggshells was conducted under the conditions of a 3-month incubation experiment in 2020. Based on the incubation experiment, an adequate object was selected with respect to the fertiliser value and its environmental management.

### Characteristics of the materials used in the incubation experiment

#### Spent coffee grounds

The applied spent coffee grounds came from one of the biggest café chains in the world (Starbucks). Spent coffee grounds were derived from a balanced blend of Arabica and Robusta beans. In accordance with the Regulation of the Minister of Climate on waste catalogue, chicken and duck eggshells have the code 20 01 08 and belong to the group 20 “Municipal wastes including separately collected fractions”. They belong to the subgroup 01, which includes “municipal wastes segregated and collected selectively”, and are of the waste type 08—“biodegradable kitchen wastes”^13^.

The collected spent coffee grounds were dried in a forced air circulation dryer at 70 °C until a constant weight was obtained. After that, they were homogenised to ensure material homogeneity and stored in linen bags in a dry, dark, and cool place. Spent coffee grounds had a very acid reaction, measured in distilled water (pH_H2O_ = 4.97), a very high carbon content, and a high content of total nitrogen. Spent coffee grounds were also rich in macronutrients, while having a very low Cd and Pb contents (Table 1).

**Table 1:** Basic physicochemical properties of spent coffee grounds and bird eggshells.

Parameter	Unit	Spent coffee grounds	Eggshells	
			Chicken	Duck
Dry matter	%	43.25 ± *2.15*	98.52 ± *4.15*	97.45 ± *3.13*
pH_H2O_	–	4.97 ± *0.10*	9.38 ± *0.10*	9.10 ± *0.10*
**Macronutrients**	**g kg**^**−1**^** DM**			
C		500.34 ± *9.70*	145.43 ± *3.07*	167.03 ± *9.66*
N		25.61 ± *1.07*	8.47 ± *0.19*	15.81 ± *2.52*
P		1.53 ± *0.04*	1.00 ± *0.04*	1.43 ± *0.05*
K		5.06 ± *0.24*	0.66 ± *0.06*	0.45 ± *0.06*
Ca		1.77 ± *0.31*	231.67 ± *3.23*	216.63 ± *6.71*
Mg		1.48 ± *0.02*	2.44 ± *0.06*	1.68 ± *0.03*
Na		0.13 ± *0.03*	0.74 ± *0.07*	1.44 ± *0.08*
S		0.87 ± *0.04*	0.78 ± *0.04*	1.34 ± *0.24*
**Heavy metals**^**a**^	**mg kg**^**−1**^** DM**			
Cd		0.01 ± *0.01*	< 0.01 ± *0.01*	< 0.01 ± *0.01*
Pb		0.19 ± *0.01*	0.09 ± *0.02*	0.11 ± *0.03*

^a^Permissible content of pollutants in agricultural lime cannot exceed Cd 8 and Pb 200 mg kg^−1^ calcium oxide (CaO)^14^.

#### Chicken and duck eggshells

The investigated chicken and duck eggshells were obtained from fresh hatching eggs coming from chicken parent stocks (‘Ross 308’ reproductive line) and Peking duck (‘Cherry Valley’ reproductive line). Chicken and duck eggshells were sourced from E.G.G. Ltd. in Poland. In accordance with the Regulation of the Minister of Climate on waste catalogue, chicken and duck eggshells with the code 02 02 99 belong to the group 02 “Wastes from agriculture, horticulture, aquaculture, fishing, forestry, food preparation and processing”. They belong to the subgroup 02, which includes “wastes from the preparation and processing of foods of animal origin”, and are of the waste type 99—“wastes not otherwise specified”^13^.

The collected chicken and duck eggshells were dried in a forced air circulation dryer at 70 °C until a constant weight was obtained. After drying, the shells were homogenised to obtain a homogeneous material. The chicken and duck eggshells prepared in this way had an alkaline reaction, measured in distilled water. The pH_H2O_ value for the chicken and duck eggshells reached 9.4 and 9.1, respectively. The chicken and duck eggshells, similarly to spent coffee grounds, contained substantial amounts of macronutrients (Table 1). The Cd and Pb contents in chicken and duck eggshells did not exceed the permissible content of these elements in calcium fertilisers^14^.

#### Scheme and course of the incubation experiment

In an incubation experiment, two types of chicken and duck eggshells were the first factor (Table 2). The date of chicken and duck eggshell collection was the second factor of the experiment. Dates of eggshell collection resulted from technological procedures in artificial incubation and were as follows: 0, 7, 18, and 21 days for chicken eggs, and 0, 9, 24, and 28 days for duck eggs, respectively (Table 2). Spent coffee grounds were mixed with chicken and duck eggshells at 10:1 ratio (200:20 g/g), (Table 2). The incubation experiment was conducted in triplicate. Within each combination, the prepared materials were placed in closed 500 cm^3^ polyethylene containers.

**Table 2:** Experimental design.

No	Object name^a^	Spent coffee grounds (SCG)	Eggshells	Dates of eggshell collection^b^	
		(g)	(g)	Chicken	Duck
1	SCG	200	–	–	–
2	CES	–	200	(0 d.i.)	–
3	DES	–	200	–	(0 d.i.)
4	SCG-CES(I)	200	20	(0 d.i.)	–
5	SCG-CES(II)	200	20	(7 d.i.)	–
6	SCG-CES(III)	200	20	(14 d.i.)	–
7	SCG-CES(IV)	200	20	(21 d.i.)	–
8	SCG-DES(I)	200	20	–	(0 d.i.)
9	SCG-DES(II)	200	20	–	(9 d.i.)
10	SCG-DES(III)	200	20	–	(19 d.i.)
11	SCG-DES(IV)	200	20	–	(28 d.i.)

^a^*SCG* spent coffee grounds, *CES* chicken eggshells, *DES* duck eggshells, *I–IV* dates of eggshell collection.

^b^0 days—prior to incubation, d.i.—day of embryo development (day of egg incubation in a hatchery).

The material in polyethylene containers was moistened with redistilled water, maintaining the substrate moisture content at 60% of the maximum water capacity, and stored at room temperature (22–23 °C) in the dark for 3 months. Once the experiment was finished, chemical analyses were carried out on the incubated material. On this basis, the chemical composition was determined in terms of assessing the possible use of these wastes for the production of organic-mineral fertilisers or soil improver.

#### Methodology of chemical analyses in waste materials

In waste materials, the following parameters were determined: the dry matter content—by weight method after drying the sample in a dryer at 105 °C^15^, and pH—potentiometrically in a suspension of the studied material and water (1:10)^16^.

The experiment consisted of 11 objects (Table 2) with three replications. Thus, 33 samples were subjected to chemical analysis, in which 0.5 g of material samples were taken from each replicate (11 objects × 3 replications).

Spent coffee grounds and chicken and duck eggshells as well as their mixtures were wet mineralised by the microwave method. Mineralisation was carried out using a mixture of concentrated HClO_4_ (70%) and HNO_3_ (65%) acids, (3:2 v/v)^17^. The content of elements (P, K, Mg, Ca, Na) in the obtained filtrates was determined using an atomic emission spectrometer—Perkin Elmer ICP-OES Optima 7300 DV^18,19^. The content of C, N, S in the waste materials and their mixtures was determined by the catalytic combustion method using the Elementar Vario Max Cube analyser.

#### Quality control of analyses

Determinations in each of the analysed samples were carried out in three replications. The accuracy of the analytical methods was verified based on certified reference materials: CRM IAEA/V—10 Hay (International Atomic Energy Agency), CRM—CD281—Rey Grass (Institute for Reference Materials and Measurements), CRM023-050—Trace Metals—Sandy Loam 7 (RT Corporation).

#### Statistical analysis

The statistical analysis of the study results was conducted using a Microsoft Office Excel 2013 spreadsheet and Statistica 13 PL package. The statistical significance of the analysed sources of variation was assessed using a two-factor analysis of variance. The significance of differences between mean values was verified by Tukey’s HSD test at the significance level of α ≤ 0.01. For selected parameters, Pearson's linear correlation coefficients (r) were computed at the significance level of α ≤ 0.01. Five percent (5%) was adopted as the maximum dispersion between measurements in the chemical analysis.

Principal component analysis (PCA) (STATISTICA 13.0, TIBCO Software Inc., Palo Alto, CA, USA) and multivariate interprofile data analysis (MS Office 2019) were performed to compare mineral profiles (not individual shares of elements). The multivariate interprofile data analysis was preceded by the unitisation of the content of minerals and pH to a nine-point scale. After that, multivariate profiles of mineral composition along with pH were established (individually for each object). The content of a given mineral in the profile was represented by its content after unitisation.

For profile comparison, Cohen’s profile similarity coefficient *r*_*c*_ was used [Cohen 1969]. This coefficient value was measured in the range from − 1.0 to 1.0, and its interpretation depended on the following values: x ≥ + 0.75 (high similarity); + 0.75 > x > + 0.30 (moderate similarity); + 0.30 ≥ x ≥ − 0.30 (no similarity); − 0.30 > x > − 0.75 (moderate dissimilarity); x ≤ − 0.75 (high dissimilarity). The closer the r_c_ values were to boundary values (1/− 1), the stronger the evaluated similarity/dissimilarity was.

## Results and discussion

**Figure 1:**
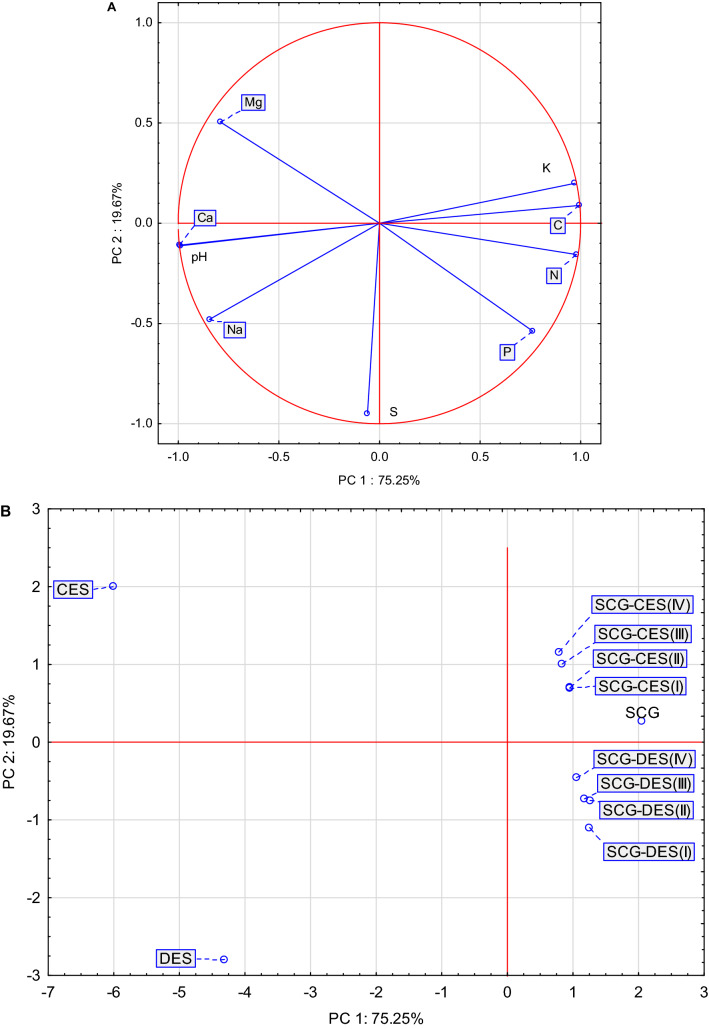
Biplot based on the first two principal component axes for the mineral composition of the mixtures (**A**) and the distribution of 11 mixtures based on the first two components obtained from the principal component analysis (**B**). Explanations in Table 2.

The chemical composition of eggshells was varied and depended on the type of eggshell (chicken, duck) and the date of eggshell collection (Table 3). The study showed that spent coffee grounds (object SCG) contained more C, N, P, and K compared to the content in the chicken and duck eggshells (objects CES, DES). Chicken and duck eggshells were richer in Ca, Mg and Na, which was also confirmed by the PCA analysis (Fig. 1). It was also observed that duck eggshells (object DES) were richer in S compared to spent coffee grounds. From the point of view of the chemical composition of these different wastes, it was established that the C, N, P, and K contents in spent coffee grounds were 3.4, 3.0, 1.5, 7.6 times higher, respectively, compared to these contents in chicken eggshells. Spent coffee grounds were also 3.0, 1.6, 1.0, 11.2 times richer in C, N, P, and K, respectively, compared to duck eggshells.

**Table 3:** Contents of macronutrients in the study objects after 3-month incubation.

Object^a^	C	N	P	K	Ca	Mg	Na	S
	g kg^−1^ DM							
**Chicken eggshells**								
SCG	500.34 ± *9.70*	25.61 ± *1.07*	1.53 ± *0.04*	5.06 ± *0.24*	1.77 ± *0.31*	1.48 ± *0.02*	0.13 ± *0.03*	0.87 ± *0.04*
CES	145.43 ± *3.07*	8.47 ± *0.19*	1.00 ± *0.04*	0.66 ± *0.06*	231.67 ± *3.23*	2.44 ± *0.06*	0.74 ± *0.07*	0.78 ± *0.04*
SCG-CES(I)	466.09 ± *8.03*	23.62 ± *0.44*	1.49 ± *0.02*	4.99 ± *0.02*	35.98 ± *3.64*	1.76 ± *0.06*	0.20 ± *0.03*	0.84 ± *0.04*
SCG-CES(II)	458.44 ± *5.75*	23.60 ± *0.71*	1.47 ± *0.06*	4.84 ± *0.27*	33.41 ± *1.06*	1.68 ± *0.06*	0.19 ± *0.01*	0.82 ± *0.06*
SCG-CES(III)	454.48 ± *7.37*	23.45 ± *1.13*	1.38 ± *0.01*	4.81 ± *0.05*	32.50 ± *0.93*	1.63 ± *0.05*	0.18 ± *0.02*	0.79 ± *0.05*
SCG-CES(IV)	442.55 ± *4.36*	22.33 ± *0.62*	1.35 ± *0.01*	4.60 ± *0.08*	27.26 ± *1.79*	1.53 ± *0.08*	0.17 ± *0.03*	0.74 ± *0.03*
Mean	411.23	21.18	1.37	4.16	60.43	1.75	0.27	0.81
CV(%)	30.1	28.2	13.4	39.0	131.9	19.0	83.1	7.2
**Duck eggshells**								
SCG	500.34 ± *9.70*	25.61 ± *1.07*	1.53 ± *0.04*	5.06 ± *0.24*	1.77 ± *0.31*	1.48 ± *0.02*	0.13 ± *0.03*	0.87 ± *0.04*
DES	167.03 ± *9.66*	15.81 ± *2.52*	1.43 ± *0.05*	0.45 ± *0.06*	216.63 ± *6.71*	1.68 ± *0.03*	1.44 ± *0.08*	1.34 ± *0.24*
SCG-DES(I)	477.17 ± *6.19*	24.74 ± *0.60*	1.50 ± *0.02*	4.86 ± *0.16*	32.27 ± *1.10*	1.54 ± *0.02*	0.25 ± *0.03*	1.26 ± *0.02*
SCG-DES(II)	474.25 ± *9.33*	23.68 ± *0.25*	1.49 ± *0.13*	4.74 ± *0.08*	28.05 ± *3.08*	1.44 ± *0.02*	0.22 ± *0.01*	1.15 ± *0.04*
SCG-DES(III)	462.65 ± *6.82*	23.49 ± *0.34*	1.44 ± *0.07*	4.27 ± *0.11*	26.59 ± *4.32*	1.32 ± *0.06*	0.21 ± *0.01*	1.13 ± *0.03*
SCG-DES(IV)	454.77 ± *4.01*	22.89 ± *0.52*	1.39 ± *0.03*	4.21 ± *0.03*	26.07 ± *1.32*	1.31 ± *0.02*	0.19 ± *0.01*	1.09 ± *0.02*
Mean	422.70	22.70	1.46	3.93	55.23	1.46	0.41	1.14
CV(%)	28.1	15.2	5.0	41.6	135.8	9.1	118.0	15.2
**Mean for object (shells)**								
SCG	500.34	25.61	1.53	5.06	1.77	1.48	0.13	0.87
CES/DES	156.23	12.14	1.22	0.56	224.15	2.06	1.09	1.06
(I)	471.63	24.18	1.49	4.93	34.13	1.65	0.22	1.05
(II)	466.35	23.64	1.48	4.79	30.73	1.56	0.21	0.98
(III)	458.57	23.47	1.41	4.54	29.54	1.48	0.19	0.96
(IV)	448.66	22.61	1.37	4.40	26.66	1.42	0.18	0.92
LSD for object^b^	6.86	0.93	0.05	0.14	2.76	0.04	0.04	0.07
LSD for shells	11.88	1.60	0.09	0.23	4.78	0.07	0.06	0.13
LSD for interaction	16.81	2.27	0.12	0.33	6.76	0.10	0.09	0.18

^a^Explanations in Table 2.

^b^*LSD* Least significant difference.

Our study confirmed that spent coffee grounds constitute a rich source of carbon compounds and nitrogen^20,21^, while chicken and duck eggshells contain lower amounts of carbon and total nitrogen. Carbon and nitrogen in spent coffee grounds occur in different organic compounds^21,22^, and the carbon present in chicken and duck eggshells occurs mainly in amino acids^6^ and in minerals such as calcium carbonates and magnesium carbonates^23^. Nitrogen in chicken and duck eggshells occurs mainly in amino acids^6,24^, which, once released to the soil environment, can be taken up by plants^25^.

Chemical composition determinations showed that objects with duck eggshells (object DES) contained more C, N, P, Na, and S compared to objects with chicken eggshells (object CES). Chicken eggshells (object CES) contained more Ca, Mg, and K compared to duck eggshells (object DES). The present study confirmed that the chemical composition of eggshells depends on the poultry species, and also on feeding, genetic features and even on different evolution processes^6,7,26^. Studies of the above-mentioned authors also indicated that chicken and duck eggshells consist mainly of calcium, in the form of calcium carbonate^7,26^, phosphorus, in the form of tricalcium phosphate, and magnesium, in the form magnesium carbonate, as well as other macro- and micronutrients^11,27,28^. The higher sulphur content in duck eggshells results from the higher content of sulphur amino acids compared to chicken eggshells^6^. The inner eggshell membranes as well as vitelline membranes of duck eggs are richer in proteins that contain sulphur amino acids^6^.

When assessing the chemical composition of only chicken and duck eggshells (objects CES, DES) with respect to their applicability as a raw material for the production of calcium fertiliser, it can be stated that chicken eggshells are more valuable due to their higher content of Ca and Mg as soil deacidifying components, and K as a fertiliser component. The requirements set in regulations concerning lime fertilisers indicate that adequate contents of calcium and magnesium in these fertilisers are a significant factor, whereas other fertiliser components can be added to these fertilisers, but they are not obligatory. Additionally, calcium fertilisers in the soil environment have a deacidifying function, are a source of calcium for plants and improve the soil structure^12,14,29,30^.

The chemical composition of the mixtures of spent coffee grounds and chicken and duck eggshells (objects SCG-CES I–IV and SCG-DES I–IV) was mainly determined by the chemical composition of spent coffee grounds. This was because the share of that waste in the mixture was 90%. In addition, the chemical composition of the above mixtures was affected by the type of shells (chicken and duck) and the date of their collection (Table 3). Ours and other studies showed that eggshells differ in chemical composition^7,31^.

The contents of Ca, Mg, and K were higher in the mixtures of spent coffee grounds and chicken eggshells (objects SCG-CES I–IV) compared to the objects with duck eggshells (SCG-DES I–IV). The higher Ca and Mg contents in those mixtures were mainly determined by the chemical composition of chicken eggshells (object CES). Ours and other studies revealed that chicken eggshells have more Ca and Mg compared to duck eggshells^26,27^. The potassium content in those mixtures came mainly from spent coffee grounds (object SCG). Spent coffee grounds, despite the high organic matter content, are a rich source of potassium and other macronutrients, which, after mineralisation, are available to plants^4,32^. The study by Cruz et al.^4^ showed that spent coffee grounds contained more nutrients than soil, which indicates that this waste can be used for the production of organic-mineral fertilisers or soil improvers^14,20,30^.

Our study showed that the mixtures of spent coffee grounds and duck eggshells (objects SCG-DES I–IV) contained more C, N, P, Na, and S compared to the mixtures with chicken eggshells. The higher content of these nutrients in the studied mixtures was determined by the higher content of these nutrients in spent coffee grounds (object SCG) and in duck eggshells (object DES). What is more, the study by Calik^7^ confirmed that sulphur in chicken and duck eggshells comes from sulphur amino acids such as methionine and cysteine, and its amount depends on the poultry species^6^.

When preparing mixtures appropriate in terms of the fertilising potential, it was established that the mixtures of spent coffee grounds and chicken eggshells (SCG-CES I–IV) are more beneficial as raw materials for the production of organic-calcium fertilisers. These mixtures contained more Ca and Mg as a deacidifying component and K as a fertiliser component. In accordance with the new EU^33^ and national^14,30^ regulations, organic matter and calcium (as a deacidifying component) are the most important components for organic-calcium fertilisers, and other components can be added or supplemented to them during ontogenesis^33^.

To generalise the effect of the addition of eggshells to spent coffee grounds on the mineral profile of the mixtures, Cohen’s similarity coefficient was used (Table 4). Of the 55 presented similarity coefficients, the vast majority (36) took values above 0.30, indicating lower or higher similarity (shades of green). Dissimilarity was observed 14 times (shades of red). Due to the substantial share of spent coffee grounds in the studied mixtures, their mineral profiles were generally very similar to these of spent coffee grounds (SCG) themselves. However, the mixtures with chicken eggshells showed a slightly higher similarity (all r_c_ > 0.900). This indicates their rather lower ability to improve the mineral profile of spent coffee grounds, perceived as a potentially valuable source of organic substance. This is also evidenced by the observed lack of similarity of the mineral profile of duck eggshells to that of mixtures containing (− 0.226 < r_c_ < 0.008). The date of eggshell collection did not modify the mineral profile of the mixtures. This is because similarities were high within the type of eggshells (chicken, duck) (all r_c_ > 0.900) Mineral profiles of mixtures containing CES were moderately similar to these of mixtures containing DES. Similar conclusions can be drawn from the PCA analysis, as it confirmed the distinctiveness of the mineral composition of spent coffee grounds and their mixtures from duck or chicken eggshells (Fig. 1A,B). Mixtures of eggshells and spent coffee grounds are a good source of organic carbon, but mixtures of chicken eggshells are richer in potassium and poorer in nitrogen compared to mixtures containing duck eggshells. It is worth underlining that the first two components explained as much as 95% of the total variance.

**Table 4:**
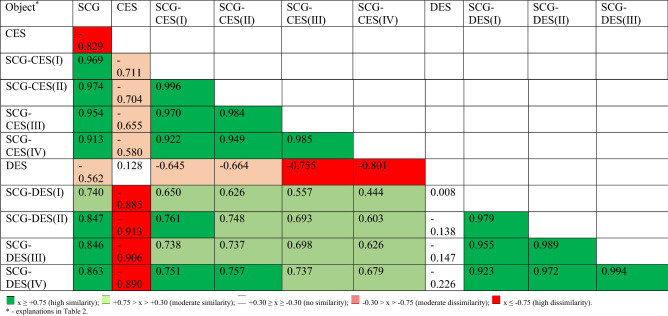
Mineral profile comparative analysis (Cohen’s profile similarity coefficient) for mixtures.

The experiment showed that the content of the studied components in the mixtures declined systematically with the date of eggshell collection (objects: SCG-CES I–IV, SCG-DES I–IV) (Table 3). The largest quantities of these nutrients were recorded on date I, and the smallest on the last date (IV). The lower content of nutrients in eggshells collected on date IV (after incubation) stems from the fact that the developing bird embryo takes all minerals required for proper development from the eggshell, yolk and white^23,34^. Other studies confirmed that from day 10–11 of incubation, the embryo uses up considerable amounts of minerals as bone formation progresses. Therefore, the calcium content decreases even by approx. 30% in the shell and yolk bag on the day of hatching^35^.

The reaction of organic waste permitted for use in agriculture is a very important parameter, because the mobility of heavy metals and their uptake by plants depend on it^36–38^. Our study showed that spent coffee grounds, after a 3-month incubation, had a very acid reaction (Table 5). Other authors’ studies also confirm that spent coffee grounds are characterised by a very acid reaction, which results from the substantial content of organic acids^3,39^. When large doses of spent coffee grounds are applied directly to the soil, this procedure will affect soil acidification and consequently increase the availability of heavy metals and their uptake by plants^40,41^. That is why spent coffee grounds, given the substantial acidification, should not be applied to soil alone, particularly in large doses and onto acid soils^39^.

**Table 5.** pH values of study objects after a 3-month incubation.

Object^a^	pH_H20_ value	Reaction^c^
**Chicken eggshells**		
SCG	4.93 ± *0.17*	Extremely acid
CES	9.28 ± *0.04*	Alkaline
SCG-CES(I)	5.96 ± *0.01*	Acid
SCG-CES(II)	5.94 ± *0.02*	Acid
SCG-CES(III)	5.81 ± *0.01*	Acid
SCG-CES(IV)	5.70 ± *0.05*	Acid
Mean	6.23	Slightly acid
CV(%)	22.9	–
**Duck eggshells**		
SCG	4.93 ± *0.17*	Extremely acid
DES	9.03 ± *0.02*	Alkaline
SCG-DES(I)	5.82 ± *0.02*	Acid
SCG-DES(II)	5.82 ± *0.01*	Acid
SCG-DES(III)	5.75 ± *0.03*	Acid
SCG-DES(IV)	5.63 ± *0.02*	Acid
Mean	6.16	Slightly acid
CV(%)	22.1	–
**Mean for object (shells)**		
SCG	4.93	Extremely acid
CES/DES	9.16	Alkaline
(I)	5.89	Acid
(II)	5.88	Acid
(III)	5.78	Acid
(IV)	5.66	Acid
LSD for object^b^	0.07	–
LSD for shells	0.12	–
LSD for interaction	0.17	–

^a^Explanations in Table 2.

^b^*LSD* Least significant difference.

^c^The reaction of fertiliser objects (mixtures) was based on the degree of soil acidity.

Our study showed that mixing acid spent coffee grounds with alkaline chicken and duck eggshells had a considerable effect on alkalisation and, in consequence, the pH value of these mixtures increased significantly (Table 5). Of the studied waste mixtures, mixture SCG-CES I had the highest applicability in agriculture. The pH value of this mixture (SCG-CES I) amounted to 5.96 and was comparable to that of biochar obtained from plant raw materials and spent coffee grounds^36,39^ and was the most useful in terms of reaction and improvement of soil physicochemical properties^20^.

Pearson’s linear correlation analysis (r) indicates a close relationship between the pH value in fertiliser mixtures and the content of Ca, Mg as deacidifying elements (r = 0.9952 and 0.7399, respectively) and Na (r = 0.8896). The study revealed that the computed correlations show a significant effect of elements in alkaline soils (Ca, Mg, Na) on the fertiliser mixture reaction.

Taking into account the date of eggshell collection and the reaction of waste mixtures for the production of organic-calcium fertiliser or soil improver, date I shells are recommended. This is because they are the richest in the deacidifying component (Ca) and other fertiliser components (Table 3). Such eggshells (of date I) can be obtained in large amounts from table egg processing plants. In addition, the chemical composition of organic-calcium fertilisers as well as soil conditioners should be stable, within specified limits, which guarantees approval for their marketing^12,14,29,30,33^.

Among all the fertiliser objects studied in the incubation experiment, the mixture of spent coffee grounds with chicken eggshells collected on date I (object SCG-CES I) was the most beneficial in terms of the content of the deacidifying component (Ca). In terms of fertiliser components such as N, P, and S, the most beneficial was the mixture of spent coffee grounds with duck eggshells, also collected on date I (SCG-DES I). The use of fresh spent coffee grounds can be toxic to plants^42^, therefore, it is proposed to compost them or treat them using another method^43^. This study showed that mixing spent coffee grounds with chicken and duck eggshells and incubating them for 3 months can give an organic-calcium fertiliser valuable to plants^39,44^, and such a mixture can also be a good soil improver.

## Conclusions

Based on the analyses carried out on the material obtained from the incubation experiment, the following conclusions were drawn:

1. Analysis of the chemical composition showed that spent coffee grounds were richer in C, N, P, and K, and chicken and duck eggshells were richer in Ca, Mg, Na, and S.
2. Compared to duck eggshells, chicken shells were richer in deacidifying components (including Ca and Mg) as well as in the fertiliser component K. Duck eggshells were richer in C, N, P, Na, and S compared to chicken eggshells.
3. The incubation experiment revealed that the content of the studied components declined systematically with the date of eggshell collection (SCG-CES I–IV, SCG-DES I–IV). The largest quantities of these nutrients were recorded in the shells of the first date, and the smallest of the last, i.e. fourth date.
4. When estimating the chemical composition and pH of objects with respect to their applicability as a raw material for the production of organic-calcium fertilisers or soil improvers, it can be concluded that mixtures of spent coffee grounds and chicken eggshells (SCG-CES I) are the most valuable because of their higher content of Ca and Mg as soil deacidifying components, and K as a fertiliser component.
5. Mixtures of spent coffee grounds and chicken eggshells can significantly improve the agricultural value of light soils with low pH, poor in organic carbon, and with poor sorption complex.
